# Introductions of Human-Origin Seasonal H3N2, H1N2 and Pre-2009 H1N1 Influenza Viruses to Swine in Brazil

**DOI:** 10.3390/v15020576

**Published:** 2023-02-19

**Authors:** Caroline Tochetto, Dennis M. Junqueira, Tavis K. Anderson, Danielle Gava, Vanessa Haach, Mauricio E. Cantão, Amy L. Vincent Baker, Rejane Schaefer

**Affiliations:** 1 Embrapa Suínos e Aves, BR 153, Km 110, Distrito de Tamanduá, Concordia 89715-899, SC, Brazil; 2 Departamento de Bioquímica e Biologia Molecular, Universidade Federal de Santa Maria (UFSM), Roraima Av., 1000, Santa Maria 97105-900, RS, Brazil; 3 Virus and Prion Research Unit, National Animal Disease Center, United States Department of Agriculture-ARS, 1920 Dayton Av., Ames, IA 50010, USA; 4 Laboratório de Virologia, Instituto de Ciências Básicas da Saúde, Universidade Federal do Rio Grande do Sul, Sarmento Leite, 500, Porto Alegre 90050-170, RS, Brazil

**Keywords:** influenza A virus, surveillance, spillover, swine, genetic diversity, reassortment

## Abstract

In South America, the evolutionary history of influenza A virus (IAV) in swine has been obscured by historically low levels of surveillance, and this has hampered the assessment of the zoonotic risk of emerging viruses. The extensive genetic diversity of IAV in swine observed globally has been attributed mainly to bidirectional transmission between humans and pigs. We conducted surveillance in swine in Brazil during 2011–2020 and characterized 107 H1N1, H1N2, and H3N2 IAVs. Phylogenetic analysis based on HA and NA segments revealed that human seasonal IAVs were introduced at least eight times into swine in Brazil since the mid-late 1980s. Our analyses revealed three genetic clades of H1 within the 1B lineage originated from three distinct spillover events, and an H3 lineage that has diversified into three genetic clades. The N2 segment from human seasonal H1N2 and H3N2 viruses was introduced into swine six times and a single introduction of an N1 segment from the human H1N1 virus was identified. Additional analysis revealed further reassortment with H1N1pdm09 viruses. All these introductions resulted in IAVs that apparently circulate only in Brazilian herds. These results reinforce the significant contributions of human IAVs to the genetic diversity of IAV in swine and reiterate the importance of surveillance of IAV in pigs.

## 1. Introduction

Influenza A virus (IAV) is one of the main respiratory pathogens in swine, affecting herds in several countries. The disease has a major economic impact for the swine industry, as well as for public health due to the emergence of IAV strains in swine with zoonotic potential. It has been argued that swine can act as a mixing vessel for human, avian, and swine IAVs that can result in pandemic IAV, and the emergence of the 2009 H1N1 pandemic virus (H1N1pdm09) reinforced this hypothesis [1]. More recent data have also revealed an intricate connection between human seasonal and endemic swine IAVs, highlighting the dramatic impact that human IAV incursion into swine populations has had in shaping the complex evolution of swine IAV (swIAV) [2,3,4,5,6,7]. Currently, a global nomenclature system has been established to classify co-circulating H1 viruses in swine defined by three major lineages: 1A, 1B, and 1C [8]. The 1A lineage is derived from viruses related to the 1918 human influenza pandemic, is colloquially known as the classical swine lineage, and includes the HA gene of the 2009 H1N1 pandemic. The 1B lineage resulted from repeated introductions of pre-2009 human seasonal H1 IAVs into swine independently in several countries, and the 1C lineage comprises viruses known as Eurasian avian-like IAV. Swine H3N2 are classified by decade of introduction of the ancestral human seasonal virus [2].

Although there is a marked genetic heterogeneity recognized among H1N1, H1N2, and H3N2 viruses circulating in pigs globally, there is limited publicly available data from Brazil, despite having the world’s third-largest swine population (approximately 37 million heads) [9]. Eighty-four percent (84%) of the swine population in Brazil is primarily found in intensive production systems located mainly in Southern, Midwestern, and Southeastern Brazil, which together account for 96.2% of pork production in the country [9]. IAV was clinically recognized in pigs in Brazil in the late 1930s, but the first viral isolation occurred only in 1974 [10]. Prior to 2009, IAV was not considered important for swine production and detection of IAV in pig herds was based only on limited serological surveys that indicated the presence of cross-reactive antibodies to the H1N1 classical swine lineage viruses and H3N2 subtype viruses [11,12,13]. Following the emergence of H1N1pdm in 2009, IAV outbreaks have become more frequently detected in Brazilian swine herds [13,14,15]. Data from swine surveillance activities in Brazil have indicated previously unappreciated genetic diversity in swine IAV as well as the persistence of novel human-to-swine incursions [16,17]. Currently, H1N1pdm09 and human-like H1N2 and H3N2 viruses circulate in swine in several Brazilian states [15,18,19,20]. A comprehensive time-scaled phylogenetic analysis revealed the introduction and circulation of three lineages of human seasonal IAV in swine that had not been previously observed in any other country [17]. These swIAVs reassorted with the H1N1pdm09 in Brazil, further increasing genetic diversity.

The objective of the present study was to identify and characterize the HA and NA genes derived from pre-2009 human seasonal viruses of H1N1, H1N2, and H3N2 IAVs isolated from pigs in Brazil from 2011 to 2020. 

## 2. Materials and Methods

### 2.1. Sample Preparation and Sequencing

**Figure 1:**
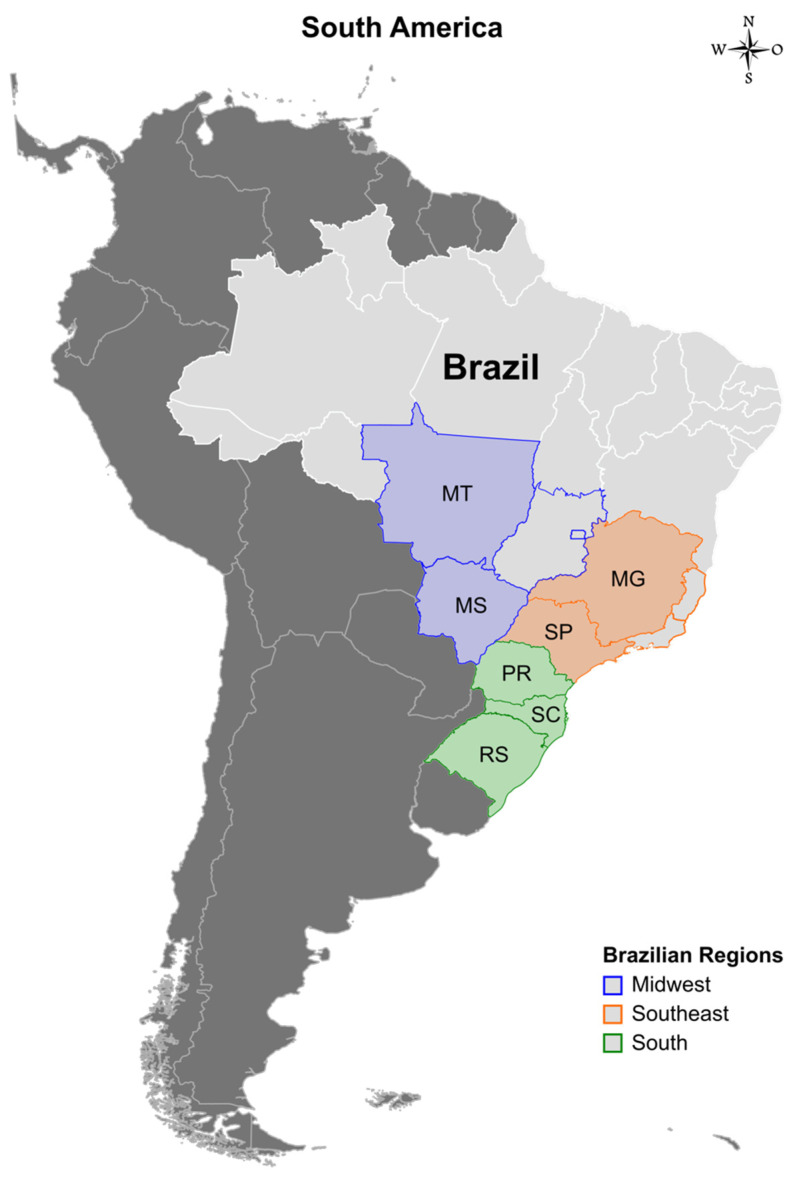
Map showing the three Brazilian regions (Midwestern, Southeastern, and Southern) and the seven states where samples were collected (color-filled): Mato Grosso (MT) with ~2.9 million pigs, Mato Grosso do Sul (MS) with ~0.69 million pigs, Minas Gerais (MG) with ~3.3 million pigs, São Paulo (SP) with ~1.3 million pigs, Paraná (PR) with ~5.8 million pigs, Santa Catarina (SC) with ~7.9 million pigs, and Rio Grande do Sul (RS) with ~6.2 million pigs.

The biological specimens (nasal swab and lung samples) were collected from suckling, nursery, and fattening pigs showing clinical signs suggestive of respiratory disease (e.g., fever, labored abdominal breathing, dyspnea, and cough) and were sent to a private diagnostic laboratory for screening of respiratory agents involved in the porcine respiratory disease complex. IAV-positive samples were sent to the Brazilian Agricultural Research Corporation (EMBRAPA) for isolation and sequencing. These samples were collected from swine between 2011 and 2020 from farms located in seven Brazilian states, representing three different regions: Rio Grande do Sul, Santa Catarina, Paraná (South), Mato Grosso do Sul, Mato Grosso (Midwest), São Paulo and Minas Gerais states (Southeast), which together account for 96.2% of pork production in the country [9] (Figure 1).

The IAV-positive samples by RT-qPCR [21] were submitted for virus isolation (VI) in SPF-embryonated chicken eggs or in MDCK cells [22]. VI was confirmed by RT-qPCR after up to two viral passages. One or two virus isolates were selected per herd for whole-genome sequencing. For sequencing, total viral RNA was extracted from allantoic fluids or cell supernatants, and the eight gene segments were amplified by RT-PCR using the following primer set: 5′-CTGGATACGCCAGCRAAAGCAGG-3′ and 5′-GACCTGATGCGGAGTAGAAACAAGG-3′ (Thermo Fisher Scientific^®^, Waltham, MA, USA). RT-PCR was run using SuperScript™ III One-Step RT-PCR System with Platinum™ Taq DNA Polymerase (Invitrogen™; Thermo Fisher Scientific^®^, Waltham, MA, USA) following the manufacture’s guidelines (PCR amplification of influenza A genomic segments for whole-genome sequencing, Ion Torrent sequencing application guide; Thermo Fisher Scientific^®^, Waltham, MA, USA). DNA libraries were prepared and submitted for sequencing using the Ion Torrent system (Thermo Fisher Scientific^®^, Waltham, MA, USA). Influenza genomes were assembled using Newbler v.2.9 (Roche, USA). In total, sequence data were obtained for 107 IAVs, including 23 partial genomes and 84 full genomes.

### 2.2. Data Set Construction

A data set was constructed for each HA and NA gene segment (H1, H3, N1, and N2). These data sets included sequences of human and swine H1N1, H1N2, and H3N2 IAVs downloaded from the Influenza Research Database (IRD) [23] and the Global Initiative on Sharing All Influenza Data (GISAID) [24], in November 2021. BLASTn was used to identify the 100 most similar HA and NA sequences available. HA H1 lineages were determined using the Swine H1 Clade Classification Tool [8]. Due to the high number of sequences in the publicly available databases and computational limitations, human IAVs were down-sampled from the data sets using CD-HIT with 98% nucleotide identity across the sequences (cd-hit-est) [25]. Phylogenetic trees were constructed with IQ-TREE2 to identify phylogenetic clades that were distantly related to Brazilian sequences and could subsequently be subsampled using CD-HIT without impacting inference. Additionally, for feasibility, we focused our analyses on HA genes from the H3 subtype and H1 1B lineage: strains containing the HA and NA of the H1N1pdm09 lineage were not included in this study and are discussed elsewhere [26]. Reference sequences used for the H1 1B phylogenetic analysis included the global reference sequences of the Swine H1 Clade Classification Tool [8]. N2 reference sequences were obtained from prior published studies describing N2 genetic diversity [27,28]. World Health Organization (WHO)-recommended human seasonal HA vaccine sequence data were also included. For our final screening, we removed duplicate gene sequences, those that were less than 1000 nt, contained “lab” or “laboratory” host metadata, or had evidence of incongruent temporal signal (assessed by root-to-tip divergence using the program TempEst v1.5.3 (Edinburgh, UK)) [29]. This sampling approach resulted in 512 H1 (1B lineage), 486 H3, 784 N2, and 150 N1 segments including: Brazilian IAV in swine gene segments obtained for this study (31 H1, 38 H3, 85 N2, and 4 N1; see Table S1); Brazilian IAV in swine sequenced and published previously, human and swine gene segments collected globally between 1930 and 2021, and WHO-recommended human seasonal HA vaccine reference sequences. The sequences obtained in this study were submitted to GenBank under the accession numbers given in Table S1.

### 2.3. Phylogenetic Analysis

Nucleotide alignments were generated separately for each gene segment dataset using MAFFT v7.490 [30] with *–leavegappyregion* and *–ep 0.123* options followed by manual correction and curation using the program AliView v1.28 (Uppsala, SE) [31]. Maximum likelihood phylogenetic trees were inferred with IQ-TREE2 (v.2.1.3) [32] using the standard, automatic best-fit model selection process (H1 and H3: TVM + F + I + G4; N2: GTR + F + I + G4; N1: K3Pu + F + G4). Statistical support was assessed using the Shimodaira–Hasegawa-like approximate Likelihood Ratio Test (SH-aLRT) [33] and Ultrafast Bootstrap approximation (UFBoot) [32,34] with 1000 replicates. A time-scaled phylogeny was inferred using TreeTime with the *–covariation* and *–confidence* parameters [35]. Final trees were visualized and edited with FigTree v.1.4.4 and Inkscape v.1.2 [36]. The estimated time of each human-to-swine transmission event is provided by the interval between two nodes on the phylogeny [37], including the confidence interval determined by the region that contains 90% of the marginal probability distribution (MPD) of the node dates. The within- and between-clade average pairwise distances were calculated using the *p-distance* method in MEGA 11 (v.11.0.13) [38]. The lineage of the internal gene segments was determined for the 84 complete genomes obtained using the *octoFLU* classifier pipeline [39].

## 3. Results

The Brazilian IAV in swine characterized here included all sequences publicly available from Brazil until 2020 (*n =* 109): one hundred and seven IAV sequences were obtained by Embrapa, some of them have been described previously [17,40], and two sequences were from another Brazilian research institution [41] Most of those are of the H1N2 subtype (64/109), followed by H3N2 (33/109), H1N1 (6/109), five H3Nx, and one H1Nx. All of the HA and NA genetic clades and their pairings described below are detailed in Table S2.

### 3.1. New Human-Origin H1N2/H1N1 in Swine

**Figure 2:**
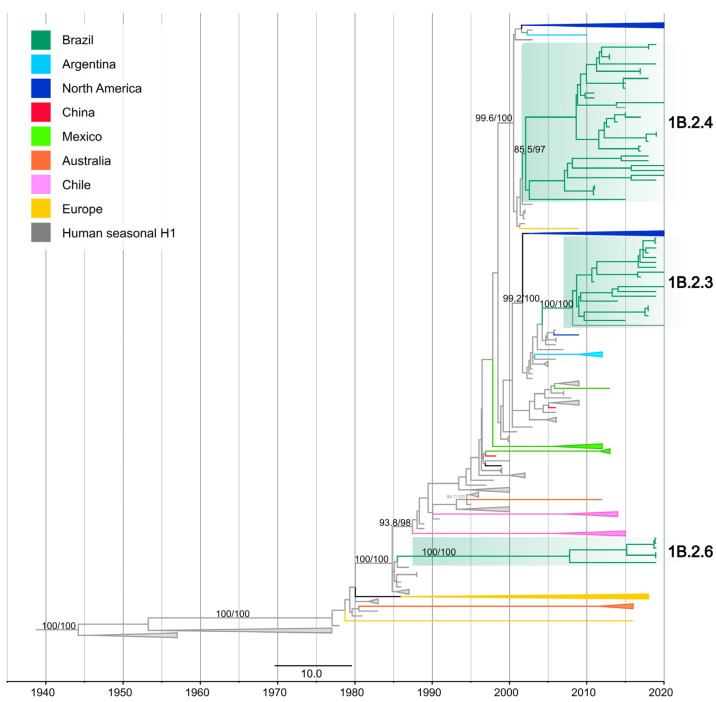
Time-scaled phylogeny demonstrating time of spillover of human seasonal H1 HA gene segments into swine in Brazil. Phylogenetic relationships of the H1 sequences from 414 human-origin H1N2 and H1N1 swine influenza virus isolates collected globally during 1939–2020, and 98 human seasonal H1N2 and H1N1 influenza viruses collected globally from humans during 1939–2020 (total dataset, *n =* 512 H1 sequences). Gray branches indicate human seasonal influenza viruses. The three clades of Brazilian swine viruses are highlighted in green (Table S2). Branches associated with viruses from swine are shaded by country of origin. Branch support values (aLRT/UF boot) > 85 are provided for key nodes.

On the H1 phylogeny, the thirty-one newly obtained H1 segments collected from swine in Brazil from 2015 to 2020 formed three statistically supported clades (SH-aLRT ≥ 85% and UFBoot ≥ 97%), representing three different introductions of the H1 segment from pre-2009 human seasonal H1N1 and H1N2 viruses into swine in Brazil. Following the swine H1 taxonomic classification proposal [8], new clade designations within the 1B lineage were proposed: 1B.2.3, 1B.2.4 [42], and 1B.2.6 (this study) (Figure 2) (Table S2).

Clade 1B.2.3 (SH-aLRT 100% and UFBoot 100%) was comprised of nineteen Brazilian swIAVs including twelve H1N2, five H1N1, one H1Nx, and one human variant (H1N2v). These isolates were collected in five Brazilian states: Rio Grande do Sul, Santa Catarina, Paraná in the South, Mato Grosso do Sul in the Midwest, and Minas Gerais in the Southeast. Clade 1B.2.3 was closely related to human seasonal H1N1 viruses that circulated during 2006–2009 (e.g., A/England/494/2006 (H1N1)). The estimated time of human-to-swine transmission was 2003.8–2008.9 (90% of MPD).

Clade 1B.2.4 (SH-aLRT 85.5% and UFBoot 97%) was comprised of thirty-three Brazilian swIAVs including thirty-one H1N2, one H1N1, and one human variant (H1N2v). These samples were collected in five Brazilian states: three in the South (Rio Grande do Sul, Santa Catarina, Paraná), and two in the Midwest (Minas Gerais and São Paulo). Clade 1B.2.4 was closely related to human seasonal H1N2 that circulated during the early 2000s (e.g., A/Memphis/8/2003 (H1N2)). The estimated time of human-to-swine transmission was 2001.2–2002.6 (90% of MPD).

Clade 1B.2.6 (SH-aLRT 100% and UFBoot 100%) was comprised of six Brazilian swIAVs, including five H1N2 and one H1N1, collected in Minas Gerais state (Southeast). Clade 1B.2.6 was closely related to human seasonal H1N1 that circulated in the late 1980s (e.g., the human seasonal vaccine strain A/Singapore/6/1986(H1N1)). The long branch length associated with this clade likely represents the lack of sampling swIAVs and only allows the time of human-to-swine transmission to be estimated broadly between 1985.1 and 2009.1 (90% of MPD). However, considering that sampling of human viruses is much more intense than in swine, the introduction was likely to have occurred closer to 1985. In addition to the strongly supported monophyletic clades, the average pairwise distance (APD) within and between these H1 clades met the criteria for the inference of new clade designations (Table S3) as follows: an APD of > 7% between clades and < 7% within the clades [8].

### 3.2. Sustained Transmission in Swine after a Single Introduction of Human H3 Segment

**Figure 3:**
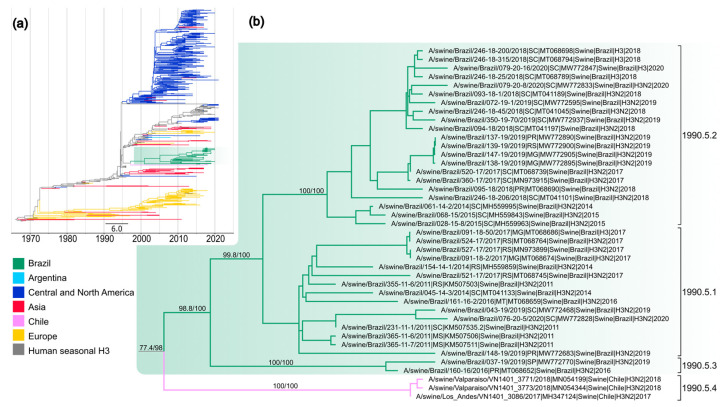
Time-scaled phylogeny demonstrating time of spillover of human seasonal H3 HA segments into swine in Brazil. (**a**) Phylogenetic relationships of the H3 sequences from 393 H3N2 swine influenza virus isolates collected globally during 1969–2021, 94 human seasonal H3N2 influenza viruses collected globally from 1968 to 2021, and 23 vaccine sequences of H3N2 from 1977 to 2020 (total dataset, *n =* 486 H3 sequences). Gray branches indicate human seasonal influenza viruses. The Brazilian clade is highlighted in green. Branches related to viruses from swine are shaded by country of origin. Branch support values (aLRT/UF boot) > 85 are provided for key nodes. (**b**) In an enlarged subtree of the 1990.5 clade, the genetic clades of H3 segments of H3N2 viruses found in swine in Brazil are identified as listed in Table S2.

All the newly sequenced H3 segments (*n =* 14) clustered with previously sequenced H3 from Brazil (*n =* 24) into a single statistically supported monophyletic clade (SH-aLRT 98.8% and UFBoot 100%), reinforced the previous findings of a single introduction of the H3 segment derived from human seasonal H3N2 viruses into Brazilian swine [17] (Figure 3).

The most closely related human seasonal virus was an H3N2 that circulated during 1996 (e.g., A/New York/596/1996 (H3N2)) and the estimated time of human-to-swine transmission was 1994.9–2001.9 (90% of MPD). This clade was included in the H3 lineage named 1990.5 [2] and shares a common ancestor with H3N2 viruses collected in pigs in Chile during 2017–2018 (Figure 3, clade 1990.5.4). The time of the most recent common ancestor of Brazilian and Chilean HA genes was 1997.8 (1996.4–1998.1 90% MPD). Considering that commercial live swine trade between Brazil and Chile is rare and the limited number of sequences from South America, the most parsimonious explanation for this finding is that human-to-swine spillover occurred during the same period of time in Brazil and Chile, but swine-to-swine transmission of a common ancestral precursor cannot be excluded. In addition, three statistically supported clades (SH-aLRT 99% and UFBoot 100%) were observed within the 1990.5 lineage, that we name here as 1990.5.1, 1990.5.2, and 1990.5.3. Consistent nucleotide distances within and between clades, with evidence for sustained onward transmission between pigs, support new genetic clade designations for the clades 1990.5.1 and 1995.5.2 (Table S4). Clade 1990.5.1 emerged early in 2008 (2007.5–2008.6 90% MDP) and contains viruses from all sampled states collected during 2011–2020. Twenty-one H3N2 swIAVs collected between 2014 and 2020 in Rio Grande do Sul, Santa Catarina, Paraná (South), and Minas Gerais (Southeast) were detected in the 1990.5.2 genetic clade. The most closely related swIAV of this clade dates 2010.3 (2009.5–2011.1 90% MDP). Clade 1990.5.3, though statistically supported (SH-aLRT/UFBoot 100%), has only two H3N2 viruses collected in Paraná in 2016 and in São Paulo in 2019, and likely represents under-sampled detection; however, it remains unknown whether H3 from this subclade persisted in swine herds until more sequences become available.

### 3.3. Novel Introductions of Human N2 Segments in Swine

**Figure 4:**
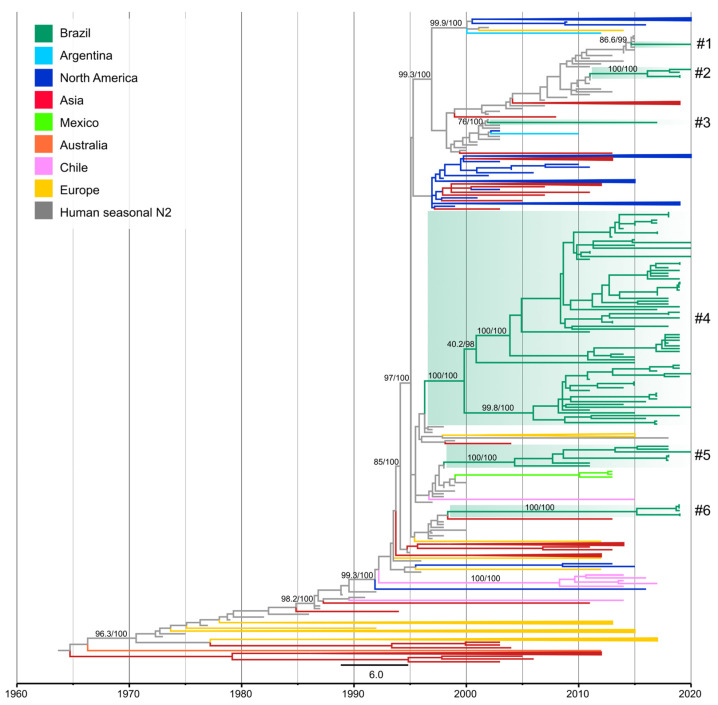
Time-scaled phylogeny demonstrating time of spillover of human seasonal N2 NA segments into swine in Brazil. Phylogenetic relationships of the N2 sequences from 704 human-origin H3N2 and H1N2 swine influenza virus isolates collected globally during 1991–2020, and 80 human seasonal H3N2 and H1N2 influenza viruses collected globally from humans during 1973–2020 (total dataset, *n =* 784 N2 sequences). Branches associated with human isolates are gray. The Brazilian clades are highlighted in green. Remaining branches related to viruses from swine are shaded by country of origin. Branch support values (aLRT/UFboot) >85 are provided for key nodes. Introductions of the segment N2 from humans into swine in Brazil are identified by the numbers with a hash “#”, as listed in Table S2.

On the N2 phylogeny, the 84 newly obtained N2 segments from H3N2 and H1N2 collected from swine between 2011 and 2020, together with previously sequenced N2 segments formed four monophyletic clades with an additional two single N2 genes nested within human N2 data. These data represent six different introductions of the N2 segment (Figure 4) from human seasonal viruses into swine. The majority of the N2 introductions (*n =* 5) were from human seasonal H3N2 with the remainder associated with a human seasonal H1N2 virus.

Clade N2-#1 (SH-aLRT 86.6 and UFBoot 99%) consisted of a single swIAV of the H1N2 subtype (A/swine/Brazil/011-20/2020) collected in Rio Grande do Sul (South). The most closely related virus was a human seasonal H3N2 virus that circulated during 2015 (e.g., A/Pelotas/LACENRS_1787/2015 (H3N2)) and the estimated time of transmission from human to swine was 2014.4–2020 (90% of MPD). This N2 segment was paired with H1 of 1B.2.3 genetic clade, which emerged in swine in the early 2000s.

Clade N2-#2 (SH-aLRT/UFBoot 100%) contained three H1N2 and one H3N2 Brazilian swIAVs (Table S2) collected in Paraná state (South) during 2019–2020. This clade was closely related to human seasonal H3N2 viruses that circulated in 2011 (e.g., A/Idaho/03/2011 (H3N2)). The estimated time of human-to-swine transmission was 2010.6–2016.8 (90% of MPD). The long branch length that separates this clade from the most closely related human virus likely indicates that this swine lineage has circulated undetected for approximately six years. Despite the low number of samples forming this clade, the statistical support for the monophyletic clade, detection across multiple years, and pairing with H1 and H3 HA genes indicates sustained onward transmission in swine. Out of the three H1N2 in this clade, one paired with an H1 1B.2.3 (A/swine/Brazil/074-20/2020/H1N2), while the other two viruses contain the HA derived from H1N1pdm09 viruses.

Clade N2-#3 (SH-aLRT 76% and UFBoot 100%) was represented by one H3N2 swIAV (A/swine/Brazil/521-17/2017) collected in Rio Grande do Sul state (South) and was closely related to human seasonal H1N2 viruses that circulated in humans during a short period of time (2001–2003) (e.g., A/North Carolina/7/2002 (H1N2)) [37]. The estimated time of human-to-swine transmission was 2001.6–2017 (90% of MPD) and this N2 segment was paired with an H3 1990.5.1 HA gene. 

Clade N2-#4 (SH-aLRT/UFBoot 100%) was the largest N2 clade detected containing seventy-three swIAVs (forty-six H1N2 and twenty-seven H3N2) and three H1N2 variants (H1N2v). These seventy-three swIAV strains were collected from the seven Brazilian states sampled during 2011–2020, revealing widespread circulation of N2 genes from this lineage. This clade was closely related to human seasonal H3N2 viruses that circulated during the late 1990s (e.g., A/Brazil/97/1997 (H3N2)) and the estimated time of human-to-swine transmission was 1995.9–2001 (90% of MPD). This estimate overlaps with the time of spillover of the H3 segment into Brazilian swine (lineage 1990.5) and likely represents the same introduction of human H3N2 viruses during the late 1990s. Of the forty-six H1N2 swIAVs within clade N2-#4, twenty-five paired with the H1 genetic clade 1B.2.4, eight paired with 1B.2.3, and thirteen H1N2 had H1 segments of the 1A H1 lineage (Table S2). This finding suggests there to be a relatively large amount of reassortment associated with this N2 genetic lineage.

Clade N2-#5 (SH-aLRT/UFBoot 100%) consisted entirely of H1N2 viruses from swine (*n =* 7) and wild boar (*n =* 1) collected between 2011 and 2020 in three Brazilian states: Rio Grande do Sul, Paraná (South), and Mato Grosso do Sul (Midwest). This clade was closely related to human seasonal H3N2 viruses that circulated during the late 1990s (e.g., A/Argentina/89/1998 (H3N2)). The estimated time of transmission of the human H3N2 viruses into swine was 1997.6–2005.4 (90% of MPD). Five of the eight H1N2 viruses from this clade were paired with H1 1B.2.4 HA genes and the other three viruses were paired with H1 1B.2.3 HA genes (Table S2).

Clade N2-#6 (SH-aLRT/UFBoot 100%) consisted of five H1N2 swIAVs collected in 2019 in Minas Gerais state (Southeast). This clade was closely related to human seasonal H3N2 viruses that circulated during the late 1990s (e.g., A/Alabama/01/1998 (H3N2)) and the estimated human-to-swine transmission date was 1997.1–2016.1 (90% of MPD). Clade N2-#6 was paired with 1B.2.6 H1 HA genes and the estimated time that the N2 gene was introduced into swine partially overlaps with the timing of introduction of the H1 segment into swine (1985.1–2009.1, 1B.2.6 clade). Thus, the H1N2 swIAVs forming this clade (#6) are likely the product of at least two human-to-swine transmission events that most likely occurred during the late 1980s for the H1 segment (from a human seasonal H1N1) and during the late 1990s for the N2 segment (from a human seasonal H3N2) followed by reassortment. The statistically supported monophyletic clade is evidence of onward transmission in pigs, although more genomic surveillance is required to better support this inference. H1N2 viruses from the N2-#6 clade share a common ancestor with H3N2 collected in 2013 in Japanese pigs (A/swine/Tochigi/14/2013). The time of the most recent common ancestor between those viruses dates to 1998.3. Considering that the movement of pigs between Brazil and Japan is not reported (https://comtradeplus.un.org/ (accessed on 2 December 2022)) and the limited number of sequences available from both countries, the most parsimonious explanation for this finding is that human-to-swine spillovers occurred during the same period in Brazil and Japan. 

### 3.4. One Introduction of Pre-2009 Human IAV N1 Segment Paired with 1B HA Genes in Swine in Brazil

**Figure 5:**
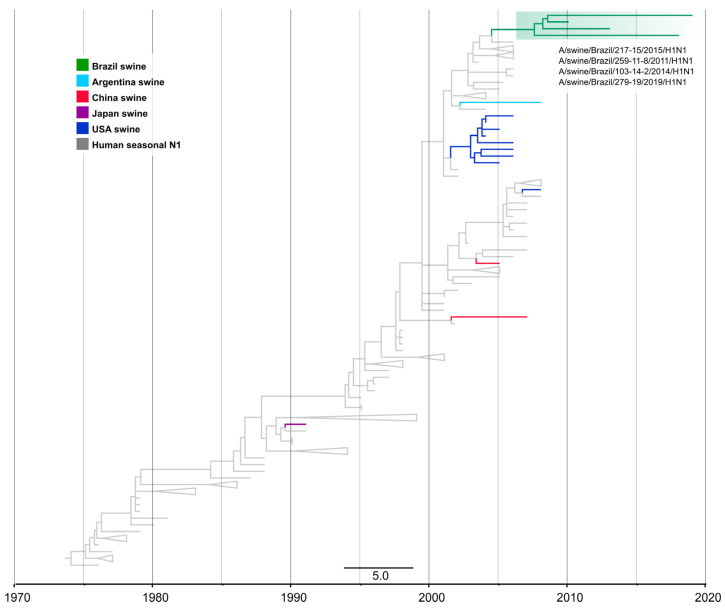
Time-scaled phylogeny demonstrating time of spillover of the human seasonal N1 NA segment into swine in Brazil. Phylogenetic relationships of the N1 sequences from 17 H1N1 swine influenza virus isolates collected globally during 1992–2019 and 133 human seasonal H1N1 influenza viruses collected globally from humans during 1977–2009 (total dataset, *n =* 150 N1 sequences). Gray branches indicate human isolates. Branches associated with viruses from swine isolates are shaded by country of origin. The Brazilian clade is highlighted in green. Branch support values (aLRT/UFboot) > 85 are provided for key nodes.

On the N1 phylogeny, the four H1N1 viruses from swine in Brazil were monophyletic, forming a single clade supported by ≥99.7% aLRT and 100% UFBoot. This clade was closely related to human seasonal H1N1 viruses that circulated during 2007 (e.g., A/Valparaiso/514/2007 (H1N1)) and was phylogenetically distinct from other swIAVs found in Argentina, North America, and Asia that are also of human seasonal H1N1 virus origin (Figure 5). 

The time-scaled tree indicates that human-to-swine spillover could have occurred between 2004.8 and 2009.3 (90% MPD). The four swIAVs H1N1 in this clade were collected in three states in Southern Brazil: Paraná, Santa Catarina, and Rio Grande do Sul. The presence of an N1 segment of human origin among samples collected during 2015–2019 together with strong branch support suggests that this segment has persisted in the swine population in Brazil, although more data are needed. All of these N1s paired with the H1 1B.2.3 lineage. The estimated time of transmission for both segments suggests that, after the introduction of a pre-2009 human H1N1 virus into swine, the N1 gene was replaced by an N2 or N1pdm, with few pre-2009 human seasonal N1 remaining, despite the H1 1B.2.3 lineage being more frequently detected with other NA.

### 3.5. Reassortment with H1N1pdm09 Viruses

The internal gene segments of the 84 full genomes evaluated using *octoFLU* classifier were revealed to be entirely of H1N1pdm09 origin (Figures S1–S6). This finding indicates reassortment among H1N1pdm09 viruses and other swIAVs of human seasonal virus origin circulating in Brazil. 

## 4. Discussion

In the current study, successful incursions and sustained transmission of distinct human seasonal H1N1, H1N2, and H3N2 IAVs into swine populations in Brazil were characterized. Long branch lengths associated with these swine HA and NA genes indicate that numerous human seasonal introductions circulated in swine for many years before being detected. Notably, new lineages of HA and NA co-circulating in Brazilian swine are related to viruses that circulated in humans more than three decades ago. These swIAVs have not been detected in any other country, corroborating with the findings of the previous study [17], although surveillance in Latin American countries is scarce. In addition, the introductions that we documented gave rise to three novel genetic clades of H1 viruses of the 1B lineage (1B.2.3, 1B.2.4, and 1B.2.6) and three novel genetic clades of H3 viruses (1990.5.1, 1990.5.2, and 1990.5.3), which are widespread in the seven Brazilian states that produce approximately 96.2% of the pork in the country [9].

At least eight distinct human-to-swine transmission events involving HA and NA segments from human seasonal influenza viruses occurred in Brazil since 1985 (Table S5). H1N1 viruses were introduced twice (in the late 1980s and in the early 2000s), H3N2 viruses were introduced five times (three times in the late 1990s, once around 2011, and once around 2015), and an H1N2 was introduced in the early 2000s. Most of the introduced HA/NA segments have become established in swine with apparently onward transmission between pigs and herds in Brazil. Now established in swine, these viruses of human-origin pose a substantial threat to swine health, and given the occasionally global movement of swine, these unique genes may spread to other segregated populations [43]. Similarly, these viruses represent a threat to public health once their antigenic segments continue to evolve in swine and become antigenically distinct from the parental viruses [44]. This risk was illustrated by the detection of H3N2v in ~300 people that attended an agricultural fair in 2012 in Ohio, U.S. [45] and other reported cases around the world [46,47,48,49]. In Brazil, zoonotic infections with IAV from swine containing surface glycoprotein genes of human seasonal ancestry were detected in 2015 [50] and 2020 [51]. In both cases, direct contact with pigs was reported since the two patients were workers at a swine farm. A recent study has demonstrated that IAVs isolated from Brazilian pigs during 2010–2018 were genetically and antigenically distinct from the contemporary human seasonal vaccine strains representing a risk to the human population due to the potential loss in population immunity against these swine IAVs [42].

All of the internal gene segments of the swine strains reported here were confirmed to be of H1N1pdm09 origin, indicating that additional reassortment events have shaped the genetic diversity of swIAVs found in Brazil. This pattern of persistence of the HA and NA segments derived from pre-2009 human seasonal viruses and replacement of the internal genes with human seasonal H1N1pdm09 through reassortment has been observed globally [4,6,37,52].

The N2 segment of H1N2 and H3N2 Brazilian swIAVs formed four genetic clades and two singletons indicating that N2 segments from H1N2 or H3N2 human seasonal viruses were introduced at least six times into swine in Brazil during the late 1990s to 2015. The N2-#4 and N2-#5 clades were previously described [17] and evidence of continually expanding genetic diversity of these clades is shown here. The most recent introduction of human seasonal N2 occurred around 2015 (clade N2-#1) and persisted until 2020, based on the sample collection date; however, more data are needed to confirm if sustained transmission between pigs has occurred since only one strain was detected in 2020. As evidence for pigs harboring genetically and potentially antigenically unique IAV genes, we detected an N2 gene in 2017 in the south of Brazil (N2-#3) and similar N2 have not been detected circulating in humans since 2003 [53].

The N1 segment of Brazilian H1N1 swIAV Isolates was classified as pre-2009 human seasonal (N1hu) or as H1N1pdm09 lineage. Detections of N1hu in swine are rare and to date were only reported in Argentina, North America, and Asia [37,54]. Although approximately 80% of the human-to-swine spillover events resulted in onward transmission of the human-origin antigenic segments (HA/NA), the onward transmission of the N1 segment of human-origin is limited in pigs [37], which may be related to the loss of viral fitness in the swine host. Nevertheless, the N1hu described here was introduced from human-to-swine around 2007 and it persisted in Brazilian swine at least until 2019 (the most recent sample collection date). In contrast to other countries, molecular detection of N1 of the classical swine lineage was not reported in Brazil until this date, although serological studies demonstrated evidence of circulation of the classical swine H1N1 lineage before 2009 [11,12,13]. 

The extent of genetic diversity of IAV detected in swine in Brazil, particularly in the HA and NA segments, is a challenge for the design of effective influenza vaccines for pigs that are currently in development [55]. Though there is no evidence of circulation of the classical, triple reassortant (TRIG) or Eurasian avian-like viruses in Brazil, we document three genetically distinct H3 HA genes, three distinct H1 1B HA lineages, and there is also evidence for the circulation of the H1 1A lineage [17]. Incorporating this many antigens into a single vaccine is likely to be challenging. Additionally, our data reveal that observed diversity is influenced by the human-to-swine transmission of novel HA genes and genomic surveillance must be maintained so that novel incursions are detected and vaccines are updated. Finally, swine breeding stock are imported from some countries of Europe and North America (https://comtradeplus.un.org/ (accessed on 2 December 2022)) and until 2014, the imported animals were kept in quarantine in farms owned by the genetic companies. Currently, imported swine are kept in a quarantine facility located on the Cananéia island in the São Paulo state supported by governmental resources, and though this process greatly reduces the likelihood of importing IAV with live swine, it does remain a possibility.

## 5. Conclusions

In summary, novel genetic clades of H1 and H3 viruses in swine were characterized. These swIAVs of human seasonal origin were circulating for over 30 years in Brazil, despite not being detected or associated with major clinical illness before 2009, when major outbreaks caused by the H1N1pdm09 virus began to arise in Brazil. The endemic swIAVs derived from these earlier human-to-swine introductions have reassorted with H1N1pdm09 and now continue to circulate in pigs in Brazil. Continued IAV surveillance and full-genome genetic characterization are critical to detect novel strains and to select representative vaccine strains to better control IAV in swine and to evaluate the risk that swine viruses pose to humans.

## Data Availability

Not applicable.

## Supplementary Materials

The following supporting information can be downloaded at: https://www.mdpi.com/article/10.3390/v15020576/s1. Figure S1: ML tree of polymerase basic (PB) 2 gene segment of the 84 full-genome Brazilian swIAVs; Figure S2: ML tree of polymerase basic (PB) 1 gene segment of the 84 full-genome Brazilian swIAVs; Figure S3: ML tree of polymerase acid (PA) gene segment of the 84 full-genome Brazilian swIAVs; Figure S4: ML tree of nucleoprotein (NP) gene segment of the 84 full-genome Brazilian swIAVs; Figure S5: ML tree of matrix (M) gene segment of the 84 full-genome Brazilian swIAVs; Figure S6: ML tree of nonstructural (NS) gene segment of the 84 full-genome Brazilian swIAVs; Table S1: List of IAV strains of the subtypes H1N1, H1N2, and H3N2 isolated in pigs in Brazil during 2011–2020 and sequenced by Embrapa; Table S2: Characteristics and phylogenetic clades of swIAV isolates in Brazilian pigs [17,41,42,43]; Table S3: Average percent pairwise nucleotide distances (*p*-distance) within and between H1 1B phylogenetic clades; Table S4: Average percent pairwise nucleotide distances (*p*-distance) within and between H3 1990.5 phylogenetic clades. Table S5. Characteristics of the eight introductions of human seasonal Influenza A viruses into swine in Brazil.
