# Supplementary material for: Introductions of Human-Origin Seasonal H3N2, H1N2 and Pre-2009 H1N1 Influenza Viruses to Swine in Brazil
Source: Viruses. 2023 Feb 19;15(2):576. doi: 10.3390/v15020576 (PMC9966956; doi:10.3390/v15020576)
Supplement: Supplementary file 1 [file viruses-15-00576-s001.zip › Figures S1-S6.pdf]

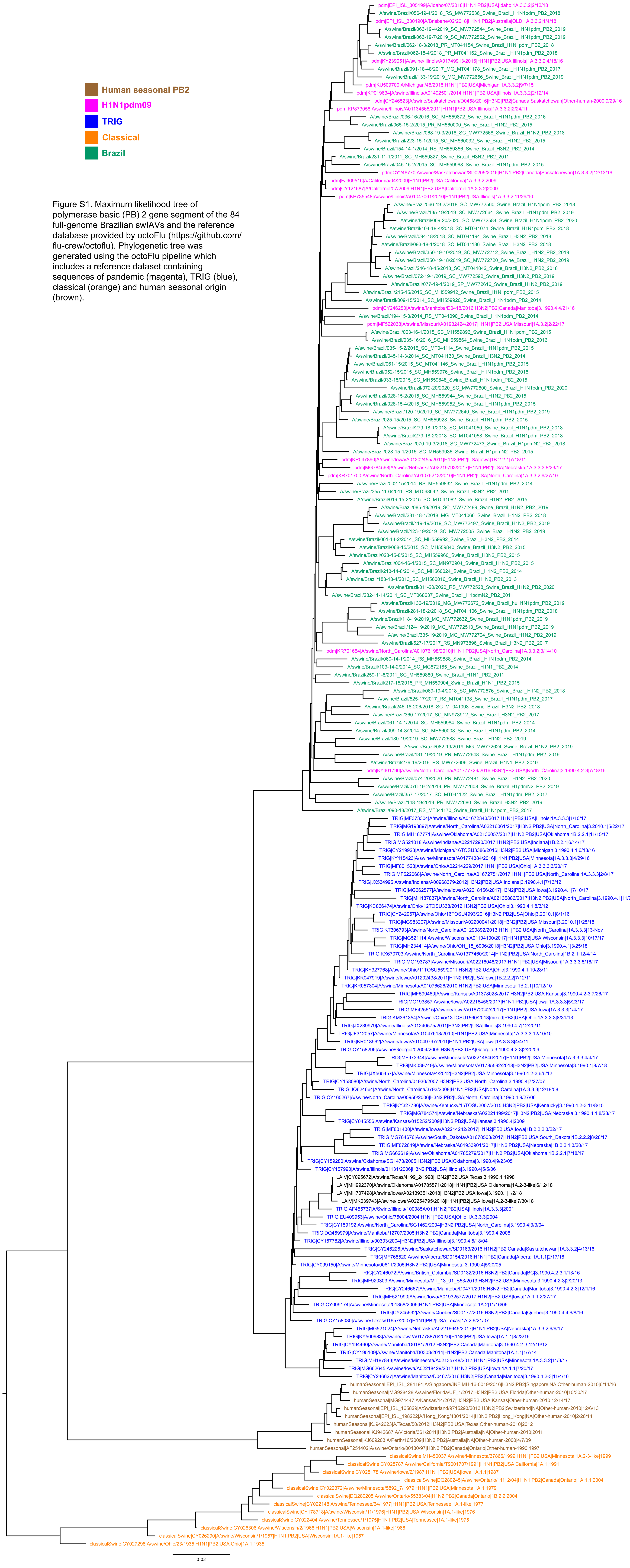

Figure S1. Maximum likelihood tree of polymerase basic (PB) 2 gene segment of the 84 full-genome Brazilian swIAVs and the reference database provided by octoFlu (https://github.com/flu-crew/octoflu). Phylogenetic tree was generated using the octoFlu pipeline which includes a reference dataset containing sequences of pandemic (magenta), TRIG (blue), classical (orange) and human seasonal origin (brown).

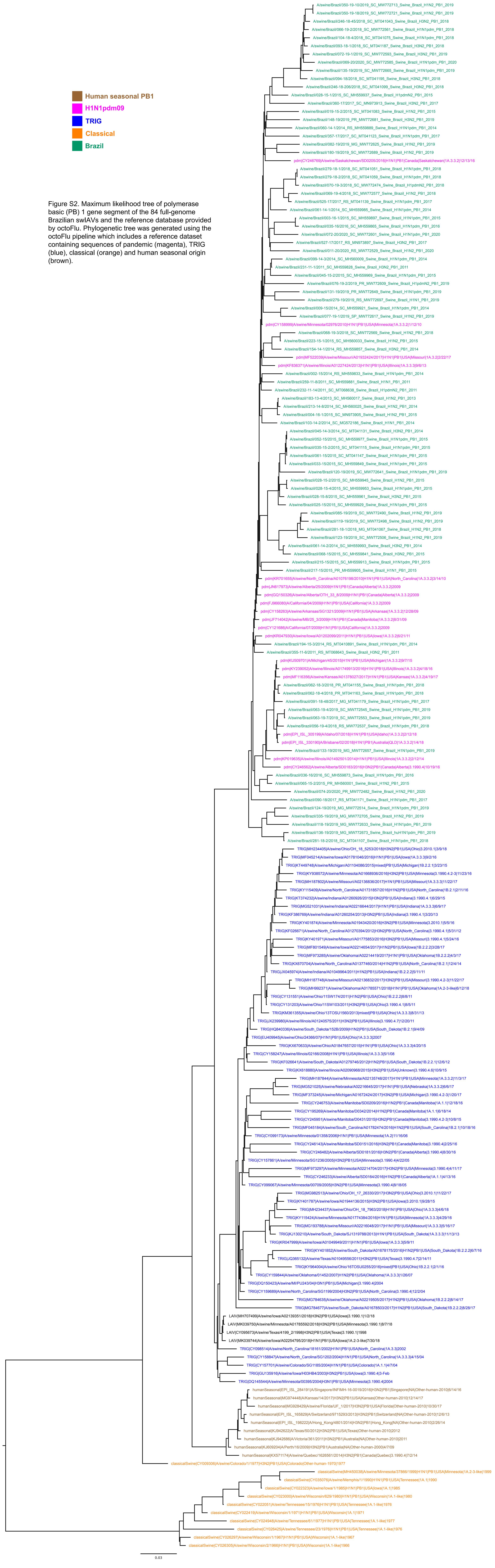

Figure S2. Maximum likelihood tree of polymerase basic (PB) 1 gene segment of the 84 full-genome Brazilian swIAVs and the reference database provided by octoFlu. Phylogenetic tree was generated using the octoFlu pipeline which includes a reference dataset containing sequences of pandemic (magenta), TRIG (blue), classical (orange) and human seasonal origin (brown).

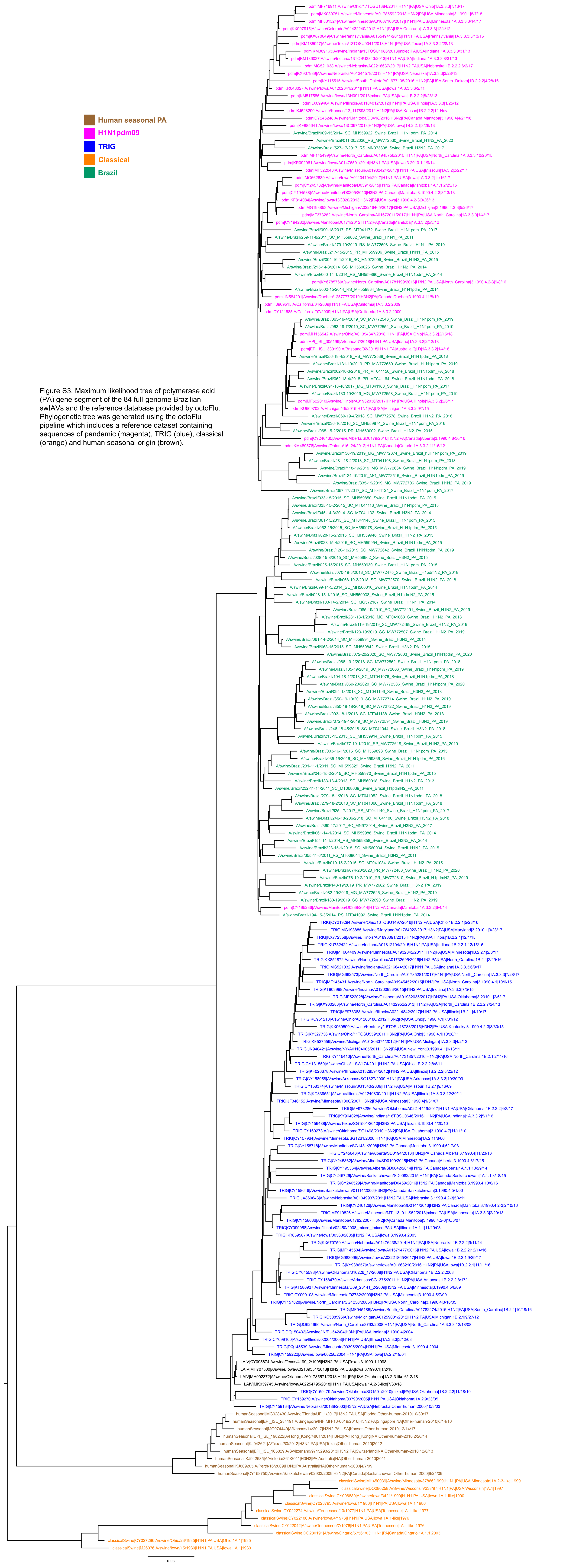

Figure S3. Maximum likelihood tree of polymerase acid (PA) gene segment of the 84 full-genome Brazilian swIAVs and the reference database provided by octoFlu. Phylogenetic tree was generated using the octoFlu pipeline which includes a reference dataset containing sequences of pandemic (magenta), TRIG (blue), classical (orange) and human seasonal origin (brown).

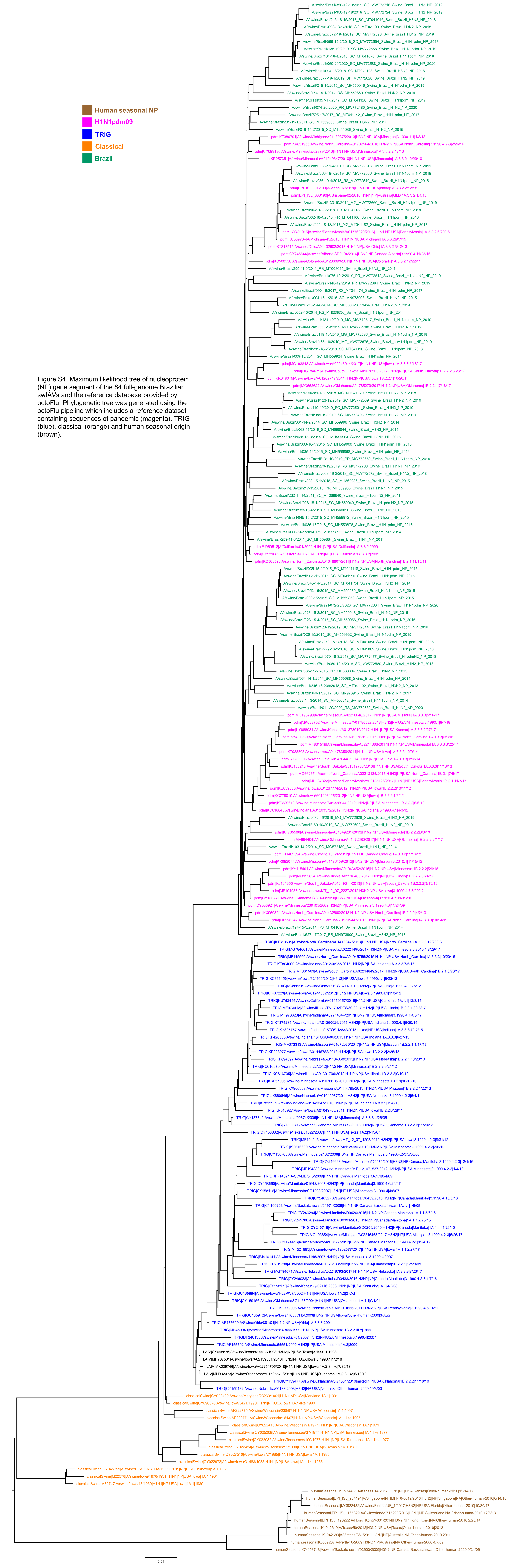

Figure S4. Maximum likelihood tree of nucleoprotein (NP) gene segment of the 84 full-genome Brazilian swIAVs and the reference database provided by octoFlu. Phylogenetic tree was generated using the octoFlu pipeline which includes a reference dataset containing sequences of pandemic (magenta), TRIG (blue), classical (orange) and human seasonal origin (brown).

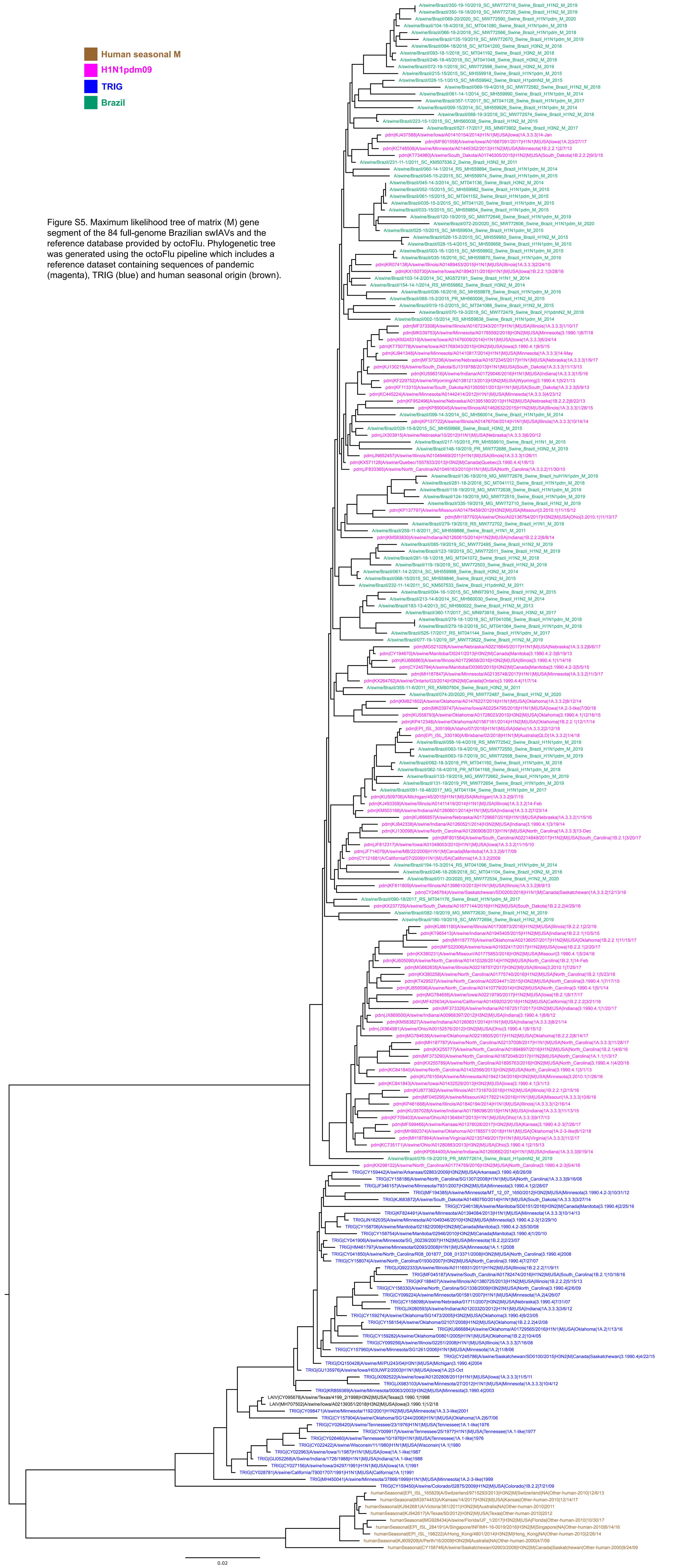

Figure S5. Maximum likelihood tree of matrix (M) gene segment of the 84 full-genome Brazilian swIAVs and the reference database provided by octoFlu. Phylogenetic tree was generated using the octoFlu pipeline which includes a reference dataset containing sequences of pandemic (magenta), TRIG (blue) and human seasonal origin (brown).

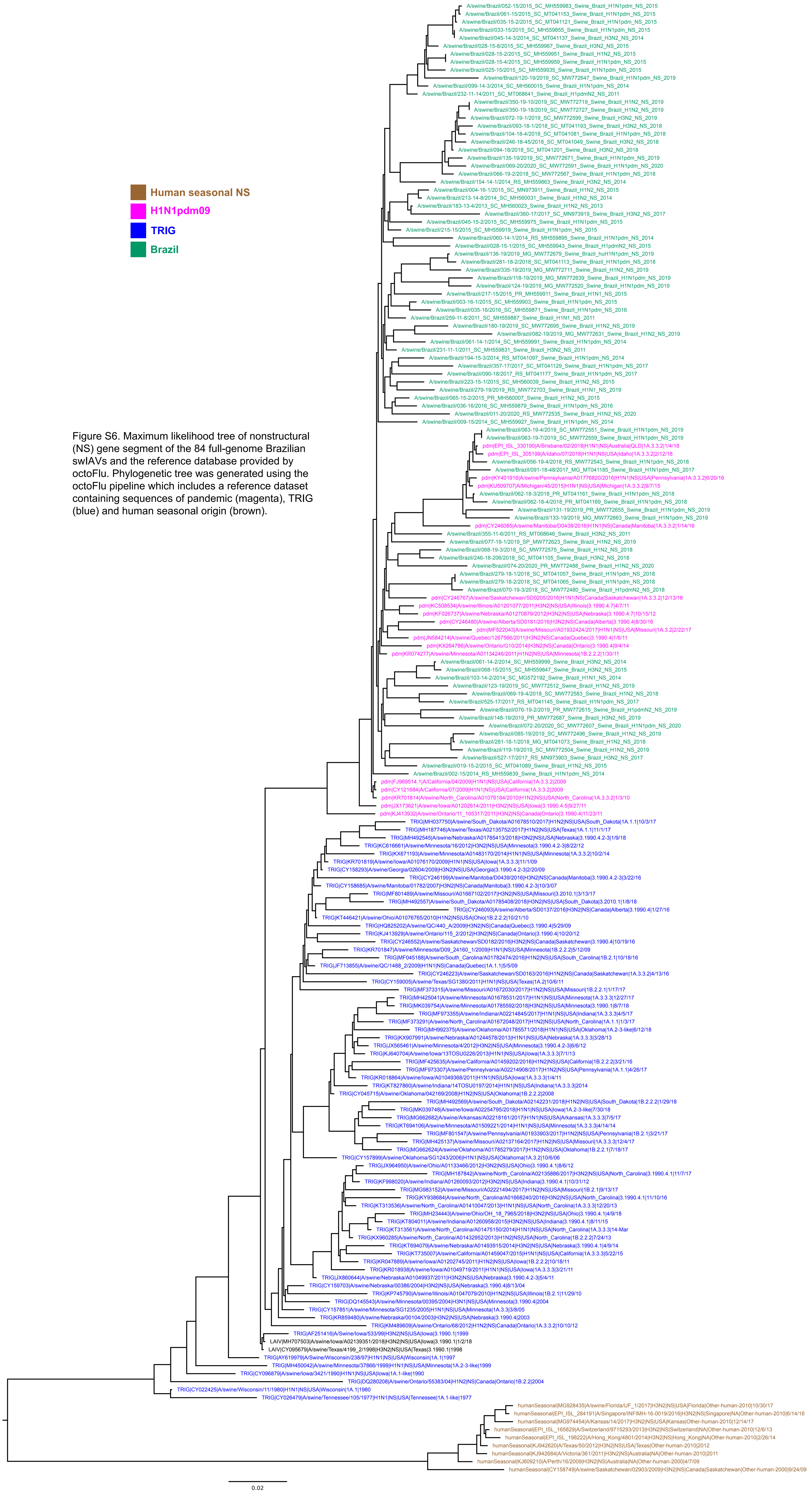

Figure S6. Maximum likelihood tree of nonstructural (NS) gene segment of the 84 full-genome Brazilian swIAVs and the reference database provided by octoFlu. Phylogenetic tree was generated using the octoFlu pipeline which includes a reference dataset containing sequences of pandemic (magenta), TRIG (blue) and human seasonal origin (brown).
