# Supplementary material for: Introductions of Human-Origin Seasonal H3N2, H1N2 and Pre-2009 H1N1 Influenza Viruses to Swine in Brazil
Source: Viruses. 2023 Feb 19;15(2):576. doi: 10.3390/v15020576 (PMC9966956; doi:10.3390/v15020576)
Supplement: Supplementary file 1 [file viruses-15-00576-s001.zip › Tables S1-S5.pdf]

Table S1. List of IAV strains of the subtypes H1N1, H1N2 and H3N2 isolated in pigs in Brazil during 2011-2020 and sequenced by Embrapa.

Virus	GenBank accession number	
	**HA**	**NA**
A/swine/Brazil/004-16-1/2015/H1N2	MN973907	MN973909
A/swine/Brazil/004-19/2019/H1N2	MW772731	MW772733
A/swine/Brazil/005-20/2019/H1N2	MW772737	MW772739
A/swine/Brazil/006-20/2019/H1N2	MW772743	MW772745
A/swine/Brazil/009-20/2020/H1N2	MW772750	MW772752
A/swine/Brazil/010-20/2020/H1N2	MW772756	MW772758
A/swine/Brazil/011-20/2020/H1N2	MW772531	MW772533
A/swine/Brazil/028-15-1/2015/H1N2	MH559939	MH559941
A/swine/Brazil/028-15-2/2015/H1N2	MH559947	MH559949
A/swine/Brazil/028-15-8/2015/H3N2	MH559963	MH559965
A/swine/Brazil/031-11-01/2011/H1N2	KF680296	KF680298
A/swine/Brazil/031-11-03/2011/H1N2	KF680291	KF680292
A/swine/Brazil/037-19/2019/H3N2	MW772770	MW772772
A/swine/Brazil/043-19/2019/H3N2	MW772468	MW772470
A/swine/Brazil/045-14-3/2014/H3N2	MT041133	MT041135
A/swine/Brazil/061-14-2/2014/H3N2	MH559995	MH559997
A/swine/Brazil/065-15-2/2015/H1N2	MH560003	MH560005
A/swine/Brazil/067-19-1/2018/H1N2	MW772776	MW772778
A/swine/Brazil/067-19-2/2018/H1N2	MW772782	MW772784
A/swine/Brazil/068-15/2015/H3N2	MH559843	MH559845
A/swine/Brazil/068-19-3/2018/H1N2	MW772571	MW772573
A/swine/Brazil/069-19-4/2018/H1N2	MW772579	MW772581
A/swine/Brazil/070-19-1/2018/H1N2	MW772789	MW772791
A/swine/Brazil/070-19-2/2018/H1N2	MW772794	MW772796
A/swine/Brazil/070-19-3/2018/H1N2	MW772476	MW772478
A/swine/Brazil/072-18-6/2018/H1N2	MT068667	MT068669
A/swine/Brazil/072-19-1/2019/H3N2	MW772595	MW772597
A/swine/Brazil/073-20/2020/H1N2	MW772808	MW772810
A/swine/Brazil/074-19-2/2019/H1N1	MW772814	MW772816
A/swine/Brazil/074-20/2020/H1N2	MW772484	MW772486
A/swine/Brazil/076-19-1/2019/H1N2	MW772821	MW772823
A/swine/Brazil/076-19-2/2019/H1N2	MW772611	MW772613
A/swine/Brazil/076-20-5/2020/H3N2	MW772828	OP785777
A/swine/Brazil/077-19-1/2019/H1N2	MW772619	MW772621
A/swine/Brazil/079-20-14/2020/H1N2	MW772840	-
A/swine/Brazil/079-20-16/2020/H3	MW772847	-
A/swine/Brazil/079-20-8/2020/H3N2	MW772833	-
A/swine/Brazil/081-19/2019/H1N2	MW772851	MW772853
A/swine/Brazil/082-19/2019/H1N2	MW772627	MW772629

A/swine/Brazil/085-19/2019/H1N2	MW772492	MW772494
A/swine/Brazil/091-18-14/2017/H1N2	MT068681	MT068683
A/swine/Brazil/091-18-2/2017/H3N2	MT068674	MT068676
A/swine/Brazil/091-18-50/2017/H3	MT068686	-
A/swine/Brazil/093-18-1/2018/H3N2	MT041189	MT041191
A/swine/Brazil/094-18/2018/H3N2	MT041197	MT041199
A/swine/Brazil/095-18/2018/H3N2	MT068690	MT068693
A/swine/Brazil/103-14-2/2014/H1N1	MG572188	MG572190
A/swine/Brazil/119-19/2019/H1N2	MW772500	MW772502
A/swine/Brazil/121-19/2019/H1N2	MW772872	MW772874
A/swine/Brazil/122-19/2019/H1N2	MW772878	MW772880
A/swine/Brazil/123-19/2019/H1N2	MW772508	MW772510
A/swine/Brazil/136-19/2019/H1N1	MW772675	MW772677
A/swine/Brazil/137-19/2019/H3N2	MW772890	MW772892
A/swine/Brazil/138-19/2019/H3N2	MW772895	MW772897
A/swine/Brazil/139-19/2019/H3N2	MW772900	MW772902
A/swine/Brazil/147-19/2019/H3N2	MW772905	MW772907
A/swine/Brazil/148-19/2019/H3N2	MW772683	MW772685
A/swine/Brazil/149-19/2019/H1N2	MW772523	MW772525
A/swine/Brazil/154-14-1/2014/H3N2	MH559859	MH559861
A/swine/Brazil/160-16/2016/H3N2	MT068652	MT068654
A/swine/Brazil/161-16-2/2016/H3N2	MT068659	MT068661
A/swine/Brazil/178-19/2019/H1N2	MW772910	MW772912
A/swine/Brazil/180-19/2019/H1N2	MW772691	MW772693
A/swine/Brazil/183-13-4/2013/H1N2	MH560019	MH560021
A/swine/Brazil/185-11-7/2011/H1N2	KM507519	KM507521
A/swine/Brazil/19-15-2/2015/H1N2	MT041085	MT041087
A/swine/Brazil/213-14-8/2014/H1N2	MH560027	MH560029
A/swine/Brazil/217-15/2015/H1N1	MH559907	MH559909
A/swine/Brazil/223-15-1/2015/H1N2	MH560035	MH560037
A/swine/Brazil/228-18-2/2018/H1N2	MT068833	MT068835
A/swine/Brazil/231-11-1/2011/H3N2	KM507535.2	KM507537.2
A/swine/Brazil/232-11-13/2011/H1N2	KM507530	KM507531
A/swine/Brazil/232-11-14/2011/H1N2	KM507532	KM507534
A/swine/Brazil/242-15-1/2015/H1N2	MT068826	MT068828
A/swine/Brazil/246-18-200/2018/H3	MT068698	-
A/swine/Brazil/246-18-206/2018/H3N2	MT041101	MT041103
A/swine/Brazil/246-18-25/2018/H3	MT068789	-
A/swine/Brazil/246-18-315/2018/H3	MT068794	-
A/swine/Brazil/246-18-45/2018/H3N2	MT041045	MT041047
A/swine/Brazil/255-19/2019/H1N2	MW772923	MW772925
A/swine/Brazil/259-11-8/2011/H1N1	MH559883	MH559885
A/swine/Brazil/274-18/2018/H1N2	MT068703	MT068705
A/swine/Brazil/276-18/2018/H1N2	MT068710	MT068712

A/swine/Brazil/279-19/2019/H1N1	MW772699	MW772701
A/swine/Brazil/281-18-1/2018/H1N2	MT041069	MT041071
A/swine/Brazil/299-17-1/2017/H1N2	MT068715	MT068717
A/swine/Brazil/299-17-2/2017/H1N2	MT068812	MT068814
A/swine/Brazil/299-17-3/2017/H1N2	MT068723	MT068725
A/swine/Brazil/334-19/2019/H1N2	MW772929	MW772931
A/swine/Brazil/335-19/2019/H1N2	MW772707	MW772709
A/swine/Brazil/350-19-10/2019/H1N2	MW772715	MW772717
A/swine/Brazil/350-19-18/2019/H1N2	MW772723	MW772725
A/swine/Brazil/350-19-70/2019/H3N2	MW772937	-
A/swine/Brazil/355-11-6/2011/H3N2	KM507503	KM507505
A/swine/Brazil/356-17/2017/H1N2	MT068819	MT068821
A/swine/Brazil/360-17/2017/H3N2	MN973915	MN973917
A/swine/Brazil/365-11-6/2011/H3N2	KM507506	-
A/swine/Brazil/365-11-7/2011/H3N2	KM507511	KM507513
A/swine/Brazil/515-17/2017/H1N2	MT068729	MT068731
A/swine/Brazil/516-17/2017/H1N2	MT068734	MT068736
A/swine/Brazil/520-17/2017/H3N2	MT068739	MT068741
A/swine/Brazil/521-17/2017/H3N2	MT068745	MT068747
A/swine/Brazil/523-17/2017/H1N2	MT068758	MT068760
A/swine/Brazil/524-17/2017/H3N2	MT068764	MT068766
A/swine/Brazil/526-17/2017/H1	MT068771	-
A/swine/Brazil/527-17/2017/H3N2	MN973899	MN973901
A/wild_boar/Brazil/214-11-13D/2011/H1N2	KF572616	KF572618

“-” NA segment not sequenced.

Table S2. Characteristics and phylogenetic clades of swIAV isolates in Brazilian pigs.

Virus	State[1]	Collection date	HA[2]	NA[3]	Reference
A/swine/Brazil/010-20/2020/H1N2	RS	2020-01-06	H1/1B.2.3	N2/#4	
A/swine/Brazil/011-20/2020/H1N2	RS	2020-01-13	H1/1B.2.3	N2/#1	
A/swine/Brazil/067-19-1/2018/H1N2	MS	2018-10-04	H1/1B.2.3	N2/#5	
A/swine/Brazil/067-19-2/2018/H1N2	MS	2018-10-04	H1/1B.2.3	N2/#5	
A/swine/Brazil/074-19-2/2019/H1N1	PR	2019-03-18	H1/1B.2.3	N1pdm	
A/swine/Brazil/085-19/2019/H1N2	SC	2019-07-05	H1/1B.2.3	N2/#4	
A/swine/Brazil/103-14-2/2014/H1N1	SC	Jun-14	H1/1B.2.3	N1‡	[43]
A/swine/Brazil/119-19/2019/H1N2	SC	2019-07-16	H1/1B.2.3	N2/#4	
A/swine/Brazil/121-19/2019/H1N2	SC	2019-07-16	H1/1B.2.3	N2/#4	
A/swine/Brazil/122-19/2019/H1N2	SC	2019-07-16	H1/1B.2.3	N2/#4	
A/swine/Brazil/123-19/2019/H1N2	SC	2019-07-16	H1/1B.2.3	N2/#4	
A/swine/Brazil/217-15/2015/H1N1	PR	2015-08-19	H1/1B.2.3	N1	
A/swine/Brazil/255-19/2019/H1N2	SC	2019-10-02	H1/1B.2.3	N2/#4	
A/swine/Brazil/259-11-8/2011/H1N1	SC	Aug-11	H1/1B.2.3	N1	[43]
A/swine/Brazil/274-18/2018/H1N2	MS	2018-04-16	H1/1B.2.3	N2/#5	[43]
A/swine/Brazil/279-19/2019/H1N1	RS	2019-10-22	H1/1B.2.3	N1	
A/swine/Brazil/281-18-1/2018/H1N2	MG	2018-07-25	H1/1B.2.3	N2/#4	[43]
A/swine/Brazil/526-17/2017/H1	RS	2017-10-04	H1/1B.2.3	-	
A/swine/Brazil/004-16-1/2015/H1N2	SC	2015-11-04	H1/1B.2.4	N2/#4	[43]
A/swine/Brazil/009-20/2020/H1N2	PR	2020-01-07	H1/1B.2.4	N2/#4	
A/swine/Brazil/31-11-01/2011/H1N2	PR	2011-03-01	H1/1B.2.4	N2/#4	[17]
A/swine/Brazil/31-11-03/2011/H1N2	PR	2011-03-01	H1/1B.2.4	N2/#5	[17]
A/swine/Brazil/068-19-3/2018/H1N2	SC	2018-10-23	H1/1B.2.4	N2/#4	
A/swine/Brazil/069-19-4/2018/H1N2	SC	2018-10-29	H1/1B.2.4	N2/#4	
A/swine/Brazil/072-18-6/2018/H1N2	RS	2018-03-21	H1/1B.2.4	N2/#5	[43]
A/swine/Brazil/073-20/2020/H1N2	PR	2020-01-28	H1/1B.2.4	N2/#5	
A/swine/Brazil/074-20/2020/H1N2	PR	2020-02-11	H1/1B.2.4	N2/#2	
A/swine/Brazil/077-19-1/2019/H1N2	SP	2019-05-28	H1/1B.2.4	N2/#4	
A/swine/Brazil/079-20-14/2020/H1N2	SC	2020-05-05	H1/1B.2.4	N2	
A/swine/Brazil/082-19/2019/H1N2	MG	2019-06-25	H1/1B.2.4	N2/#4	
A/swine/Brazil/091-18-14/2017/H1N2	MG	2017-09-25	H1/1B.2.4	N2/#4	[43]
A/swine/Brazil/180-19/2019/H1N2	SC	2019-08-20	H1/1B.2.4	N2/#4	
A/swine/Brazil/183-13-4/2013/H1N2	SC	Dec-13	H1/1B.2.4	N2/#4	[43]
A/swine/Brazil/185-11-7/2011/H1N2	SC	2011-07-06	H1/1B.2.4	N2/#4	[17]
A/swine/Brazil/19-15-2/2015/H1N2	SC	2015-02-11	H1/1B.2.4	N2/#4	[43]
A/swine/Brazil/213-14-8/2014/H1N2	SC	2014-11-03	H1/1B.2.4	N2/#4	[43]

A/swine/Brazil/223-15-1/2015/H1N2	SC	2015-10-20	H1/1B.2.4	N2/#4	[43]
A/swine/Brazil/228-18-2/2018/H1N2	MG	2018-01-19	H1/1B.2.4	N2/#4	[43]
A/swine/Brazil/232-11-13/2011/H1N2	SC	2011-08-17	H1/1B.2.4	N2/#4	[17]
A/swine/Brazil/242-15-1/2015/H1N2	SC	2015-11-05	H1/1B.2.4	N2/#4	[43]
A/swine/Brazil/276-18/2018/H1N2	PR	2018-04-23	H1/1B.2.4	N2/#5	[43]
A/swine/Brazil/299-17-3/2017/H1N2	SC	2017-08-10	H1/1B.2.4	N2/#4	[43]
A/swine/Brazil/350-19-10/2019/H1N2	SC	2019-06-04	H1/1B.2.4	N2/#4	
A/swine/Brazil/350-19-18/2019/H1N2	SC	2019-06-04	H1/1B.2.4	N2/#4	
A/swine/Brazil/515-17/2017/H1N2	MG	2017-06-05	H1/1B.2.4	N2/#4	[43]
A/swine/Brazil/516-17/2017/H1N2	MG	2017-06-06	H1/1B.2.4	N2/#4	[43]
A/swine/Brazil/523-17/2017/H1N2	SC	2017-09-11	H1/1B.2.4	N2/#4	[43]
A/swine/Brazil/G2P1/2013/H1N2	RS	2013-06-05	H1/1B.2.4	N2/#4	[42]
A/swine/Brazil/G2P2/2013/H1N2	RS	2013-06-05	H1/1B.2.4	N2/#4	[42]
A/wild_boar/Brazil/214-11-13D/2011/H1N2	RS	Aug-11	H1/1B.2.4	N2/#5	[17,41]
A/swine/Brazil/081-19/2019/H1N2	MG	2019-04-02	H1/1B.2.6	N2/#6	
A/swine/Brazil/136-19/2019/H1N1	MG	2019-08-07	H1/1B.2.6	N1‡	
A/swine/Brazil/149-19/2019/H1N2	MG	2019-09-03	H1/1B.2.6	N2/#6	
A/swine/Brazil/178-19/2019/H1N2	MG	2019-09-03	H1/1B.2.6	N2/#6	
A/swine/Brazil/334-19/2019/H1N2	MG	2019-11-11	H1/1B.2.6	N2/#6	
A/swine/Brazil/335-19/2019/H1N2	MG	2019-11-11	H1/1B.2.6	N2/#6	
A/swine/Brazil/004-19/2019/H1N2	SC	2019-01-22	H1†	N2/#4	
A/swine/Brazil/005-20/2019/H1N2	SC	2019-11-13	H1†	N2/#4	
A/swine/Brazil/006-20/2019/H1N2	SC	2019-11-13	H1†	N2/#4	
A/swine/Brazil/028-15-1/2015/H1N2	SC	2015-02-26	H1†	N2/#4	
A/swine/Brazil/028-15-2/2015/H1N2	SC	2015-02-26	H1†	N2/#4	
A/swine/Brazil/065-15-2/2015/H1N2	PR	2015-04-07	H1†	N2/#4	
A/swine/Brazil/070-19-1/2018/H1N2	SC	2018-11-21	H1†	N2/#4	
A/swine/Brazil/070-19-2/2018/H1N2	SC	2018-11-21	H1†	N2/#4	
A/swine/Brazil/070-19-3/2018/H1N2	SC	2018-11-21	H1†	N2/#4	
A/swine/Brazil/076-19-1/2019/H1N2	PR	2019-06-04	H1†	N2/#2	
A/swine/Brazil/076-19-2/2019/H1N2	PR	2019-06-04	H1†	N2/#2	
A/swine/Brazil/232-11-14/2011/H1N2	SC	2011-08-17	H1†	N2/#4	[17]
A/swine/Brazil/299-17-1/2017/H1N2	SC	2017-08-10	H1†	N2/#4	
A/swine/Brazil/299-17-2/2017/H1N2	SC	2017-08-10	H1†	N2/#4	
A/swine/Brazil/356-17/2017/H1N2	SC	2017-08-14	H1†	N2/#4	
A/swine/Brazil/043-19/2019/H3N2	SC	2019-04-24	H3/1990.5.1	N2/#4	
A/swine/Brazil/045-14-3/2014/H3N2	SC	Mar-14	H3/1990.5.1	N2/#4	[43]
A/swine/Brazil/076-20-5/2020/H3N2	SC	2020-04-14	H3/1990.5.1	N2#4	
A/swine/Brazil/091-18-2/2017/H3N2	MG	2017-09-25	H3/1990.5.1	N2/#4	

A/swine/Brazil/091-18-50/2017/H3	MG	2017-09-25	H3/1990.5.1	-	[43]
A/swine/Brazil/148-19/2019/H3N2	PR	2019-08-27	H3/1990.5.1	N2/#2	
A/swine/Brazil/154-14-1/2014/H3N2	RS	Aug-14	H3/1990.5.1	N2/#4	
A/swine/Brazil/161-16-2/2016/H3N2	MT	2016-09-09	H3/1990.5.1	N2/#4	[43]
A/swine/Brazil/231-11-1/2011/H3N2	SC	2011-08-17	H3/1990.5.1	N2/#4	[17]
A/swine/Brazil/355-11-6/2011/H3N2	RS	2011-10-27	H3/1990.5.1	N2/#4	[17]
A/swine/Brazil/365-11-6/2011/H3N2	MS	Nov-11	H3/1990.5.1	-	[17]
A/swine/Brazil/365-11-7/2011/H3N2	MS	2011-11-10	H3/1990.5.1	N2/#4	[17]
A/swine/Brazil/521-17/2017/H3N2	RS	2017-09-28	H3/1990.5.1	N2/#3	[43]
A/swine/Brazil/524-17/2017/H3N2	RS	2017-10-16	H3/1990.5.1	N2/#4	[43]
A/swine/Brazil/527-17/2017/H3N2	RS	2017-09-28	H3/1990.5.1	N2/#4	[43]
A/swine/Brazil/028-15-8/2015/H3N2	SC	2015-02-26	H3/1990.5.2	N2/#4	[43]
A/swine/Brazil/061-14-2/2014/H3N2	SC	Apr-14	H3/1990.5.2	N2/#4	
A/swine/Brazil/068-15/2015/H3N2	SC	2015-04-14	H3/1990.5.2	N2/#4	
A/swine/Brazil/072-19-1/2019/H3N2	SC	2019-01-17	H3/1990.5.2	N2/#4	
A/swine/Brazil/079-20-16/2020/H3	SC	2020-05-05	H3/1990.5.2	-	
A/swine/Brazil/079-20-8/2020/H3N2	SC	2020-05-05	H3/1990.5.2	-	
A/swine/Brazil/093-18-1/2018/H3N2	SC	2018-01-09	H3/1990.5.2	N2/#4	[43]
A/swine/Brazil/094-18/2018/H3N2	SC	2018-01-30	H3/1990.5.2	N2/#4	[43]
A/swine/Brazil/095-18/2018/H3N2	PR	2018-02-07	H3/1990.5.2	N2/#4	[43]
A/swine/Brazil/137-19/2019/H3N2	PR	2019-07-22	H3/1990.5.2	N2/#4	
A/swine/Brazil/138-19/2019/H3N2	MG	2019-07-16	H3/1990.5.2	N2/#4	
A/swine/Brazil/139-19/2019/H3N2	RS	2019-07-10	H3/1990.5.2	N2/#4	
A/swine/Brazil/147-19/2019/H3N2	MG	2019-08-13	H3/1990.5.2	N2/#4	
A/swine/Brazil/246-18-200/2018/H3	SC	2018-07-10	H3/1990.5.2	-	
A/swine/Brazil/246-18-206/2018/H3N2	SC	2018-07-24	H3/1990.5.2	N2/#4	[43]
A/swine/Brazil/246-18-25/2018/H3	SC	2018-06-28	H3/1990.5.2	-	[43]
A/swine/Brazil/246-18-315/2018/H3	SC	2018-08-16	H3/1990.5.2	-	[43]
A/swine/Brazil/246-18-45/2018/H3N2	SC	2018-07-03	H3/1990.5.2	N2/#4	[43]
A/swine/Brazil/350-19-70/2019/H3N2	SC	2019-05-06	H3/1990.5.2	-	
A/swine/Brazil/360-17/2017/H3N2	SC	2017-08-30	H3/1990.5.2	N2/#4	[43]
A/swine/Brazil/520-17/2017/H3N2	SC	2017-08-30	H3/1990.5.2	N2/#4	[43]
A/swine/Brazil/037-19/2019/H3N2	SP	2019-03-18	H3/1990.5.3	N2/#4	
A/swine/Brazil/160-16/2016/H3N2	PR	2016-09-09	H3/1990.5.3	N2/#4	[43]

HA, hemagglutinin; NA neuraminidase; MG, Minas Gerais; MS, Mato Grosso do Sul; MT, Mato Grosso; PR, Paraná; RS, Rio Grande do Sul; SC, Santa Catarina; SP, São Paulo; NI, not informed; -, sequence not available.
[1]Location of virus collection among the seven States in Southern, Midwestern and Southeastern Brazil provided for each sample (Figure 1).
[2]Genetic clade of HA sequence.
[3]Genetic clade of NA sequence.
[†]HA of 1A.3.3.2 lineage (or pandemic H1).
[‡]NA of pandemic N1.

Table S3. Average percent pairwise nucleotide distances (*p*-distance) within and between H1 1B phylogenetic clades.

	**APD (%)**													
		**Between clade**												
**Clade**	**Within clade**	**1B.1**	**1B.1.1**	**1B.1.2**	**1B.1.2.1**	**1B.1.2.2**	**1B.1.2.3**	**1B.2.1**	**1B.2.2**	**1B.2.2.1**	**1B.2.2.2**	**1B.2.3**	**1B.2.4**	**1B.2.6**
1B.1	4.7													
1B.1.1	7.3	9.2												
1B.1.2	7.3	8.9	11.4											
1B.1.2.1	5.7	8.9	11.1	8.8										
1B.1.2.2	5.9	10.4	12.3	9.9	9.5									
1B.1.2.3	5.3	9.5	11.3	8.8	8.7	9.2								
1B.2.1	5.6	13.2	14.8	15.2	15.0	16.4	14.9							
1B.2.2	3.8	11.3	13.0	13.2	13.1	14.4	12.9	8.6						
1B.2.2.1	4.4	13.6	15.0	15.6	15.3	16.3	15.2	11.2	6.7					
1B.2.2.2	4.7	12.9	14.3	14.8	14.6	15.5	14.4	10.7	6.1	6.7				
1B.2.3	5.0	12.1	13.9	14.3	14.2	15.5	14.1	8.7	7.5	10.3	9.8			
1B.2.4	6.4	12.7	14.5	14.5	14.3	15.7	14.4	10.0	7.2	9.8	9.2	9.2		
1B.2.6	3.4	14.4	15.5	15.8	15.8	17.2	15.7	15.1	13.5	15.3	14.8	14.8	14.6	

Table S4. Average percent pairwise nucleotide distances (*p*-distance) within and between H3 1990.5 phylogenetic clades.

	APD (%)				
		Between clades			
H3 Clade	Within clade	1990.5.1	1990.5.2	1990.5.3	1990.5.4
1990.5.1	4.5				
1990.5.2	2.9	7.8			
1990.5.3	1.7	9.4	10.4		
1990.5.4	0.5	11.5	12.0	11.2	

Table S5. Characteristics of the eight introductions of human seasonal Influenza A viruses into swine in Brazil.

Human-to-swine spillover event	Virus subtype	Related human seasonal IAV	Segment	Estimated interval of human-to-swine transmission (90% MPD)	Year of isolation of the most recent swIAV	Swine genetic clade	Detected IAV subtypes
1	H1N1	A/Singapore/6/1986 (H1N1)	H1	1985.1 - 2009.1	2019	1B.2.6	H1N1, H1N2
2	H1N1	A/England/494/2006 (H1N1)	H1	2003.8 - 2008.9	2020	1B.2.3	H1N1, H1N2
		A/Valparaiso/514/2007 (H1N1)	N1	2004.8 - 2009.3	2019	N1	H1N1
3	H1N2	A/Memphis/8/2003 (H1N2)	H1	2001.2 - 2002.6	2020	1B.2.4	H1N1, H1N2
		A/North Carolina/7/2002 (H1N2)	N2	2001.6 - 2017[1]	2017	N2-#3	H3N2
4	H3N2	A/NewYork/596/1996 (H3N2)	H3	1994.9 - 2001.9	2020	H3.1990.5	H3N2
		A/Brazil/97/1997 (H3N2)	N2	1995.9 - 2001	2020	N2-#4	H1N2, H3N2
5	H3N2	A/Argentina/89/1998 (H3N2)	N2	1997.6 - 2005.4	2020	N2-#5	H1N2
6	H3N2	A/Alabama/01/1998 (H3N2)	N2	1997.1 - 2016.1	2019	N2-#6	H1N2
7	H3N2	A/Idaho/03/2011 (H3N2)	N2	2010.6 - 2016.8	2020	N2-#2	H1N2, H3N2
8	H3N2	A/Pelotas/LACENRS_1787/2015 (H3N2)	N2	2014.4 - 2020[1]	2020	N2-#1	H1N2

[1] MDP, marginal probability distribution.

[2] Upper value based on the year of isolation of the single representative virus strain for this clade.
